# Reply to Nguyen et al. Over-Intake of Plant-Derived Antioxidants, H_2_S Generation, and Reductive Stress. Comment on “Manzano-Pech et al. The Chronic Elevated Consumption of *Hibiscus sabdariffa* Linnaeus Results in Kidney Damage Associated with Excess H_2_S. *Int. J. Mol. Sci.* 2026, *27*, 2190”

**DOI:** 10.3390/ijms27104286

**Published:** 2026-05-12

**Authors:** Linaloe Manzano-Pech, María Elena Soto, Vicente Castrejón-Tellez, Elizabeth Soria-Castro, Verónica Guarner-Lans, Sara Caballero-Chacón, Raúl Martínez-Memije, Juan Carlos Torres-Narváez, Mohammed El-Hafidi, Israel Pérez-Torres

**Affiliations:** 1 Department of Physiology and Pharmacology UNAM, Facultad de Medicina y Veterinaria y Zootecnia, Av. Universidad 3000, Coyoacán 04510, Mexico; loe_mana@hotmail.com (L.M.-P.); saracachas@hotmail.com (S.C.-C.); 2 Research Direction, Instituto Nacional de Cardiología Ignacio Chávez, Juan Badiano 1, Sección XVI, Tlalpan, Mexico City 14080, Mexico; mesoto50@hotmail.com; 3 Department of Physiology, Instituto Nacional de Cardiología Ignacio Chávez, Juan Badiano 1, Sección XVI, Tlalpan, Mexico City 14080, Mexico; vicente.castrejon@cardiologia.org.mx (V.C.-T.); veronica.guarner@cardiologia.org.mx (V.G.-L.); 4 Department of Cardiovascular Biomedicine, Instituto Nacional de Cardiología Ignacio Chávez, Juan Badiano 1, Sección XVI, Tlalpan, Mexico City 14080, Mexico; elizabeth.soria@cardiologia.org.mx (E.S.-C.); medelhafidi@yahoo.com (M.E.-H.); 5 Department de Electromechanical Instrumentation, Instituto Nacional de Cardiología Ignacio Chávez, Juan Badiano 1, Sección XVI, Tlalpan, Mexico City 14080, Mexico; raulmmemije@yahoo.com; 6 Department de Pharmacology, Instituto Nacional de Cardiología Ignacio Chávez, Juan Badiano 1, Sección XVI, Tlalpan, Mexico City 14080, Mexico; juancarlostn63@hotmail.com

The response to the comment regarding our article is accurate: chronic consumption of a 6% *Hibiscus sabdariffa* Linnaeus (HSL) infusion for one month in healthy rats reduces reactive oxygen species (ROS) [1,2]. Several studies by our research group on metabolic syndrome pathologies have demonstrated that HSL infusions at low concentrations (2–3%) serve as an effective alternative treatment to mitigate hypertension, insulin resistance, obesity, and hypertriglyceridemia [3,4]. This therapeutic benefit is primarily due to the modulation of oxidative damage underlying these conditions. However, chronic and excessive consumption of HSL in clinically healthy subjects can trigger adverse physiological effects. Our data indicate that the excess of antioxidant molecules from HSL overstimulates both enzymatic and non-enzymatic antioxidant systems, increasing reducing equivalents and inducing a state of reductive stress [1,5]. Furthermore, ongoing investigations are exploring whether extended exposure to 6% HSL beyond one month may extend this reductive stress pathology to other high-energy-demand organs, such as the myocardium.

The main findings regarding this model to date are:

Systemic impact: Excessive and chronic consumption of HSL infusions (3% and 6%) increases total antioxidant capacity (TAC), which paradoxically promotes a pro-inflammatory state associated with hypertension. These deleterious effects are dose-dependent and more pronounced at 6% [5].

Vascular function: The over-ingestion of HSL-derived antioxidants—such as polyphenols, quercetin, and various organic acids—may excessively deplete ROS, thereby deteriorating vascular reactivity in healthy rats [6].

Renal function: A 6% HSL infusion results in a state of reductive stress that increases GSH/GSSG and redox ratios. This imbalance impairs kidney function, although the condition appears to be reversible upon discontinuation of HSL intake [5,7].

Chronic 6% HSL consumption increases cysteine availability, inducing the overexpression of cystathionine-β-synthase (CBS) and cystathionine-γ-lyase (CSE) via Nrf2 upregulation. This “feed-forward” mechanism rises to supraphysiological levels, directly contributing to the shift toward a reductive redox environment [5].
